# No rationale for 1 variable per 10 events criterion for binary logistic regression analysis

**DOI:** 10.1186/s12874-016-0267-3

**Published:** 2016-11-24

**Authors:** Maarten van Smeden, Joris A. H. de Groot, Karel G. M. Moons, Gary S. Collins, Douglas G. Altman, Marinus J. C. Eijkemans, Johannes B. Reitsma

**Affiliations:** 1 Julius Center for Health Sciences and Primary Care, University Medical Center Utrecht, Heidelberglaan 100, Utrecht, The Netherlands; 2 Centre for Statistics in Medicine, Botnar Research Centre, University of Oxford, Oxford, UK

**Keywords:** EPV, Bias, Separation, Logistic regression, Sample size, Simulations

## Abstract

**Background:**

Ten events per variable (EPV) is a widely advocated minimal criterion for sample size considerations in logistic regression analysis. Of three previous simulation studies that examined this minimal EPV criterion only one supports the use of a minimum of 10 EPV. In this paper, we examine the reasons for substantial differences between these extensive simulation studies.

**Methods:**

The current study uses Monte Carlo simulations to evaluate small sample bias, coverage of confidence intervals and mean square error of logit coefficients. Logistic regression models fitted by maximum likelihood and a modified estimation procedure, known as Firth’s correction, are compared.

**Results:**

The results show that besides EPV, the problems associated with low EPV depend on other factors such as the total sample size. It is also demonstrated that simulation results can be dominated by even a few simulated data sets for which the prediction of the outcome by the covariates is perfect (‘separation’). We reveal that different approaches for identifying and handling separation leads to substantially different simulation results. We further show that Firth’s correction can be used to improve the accuracy of regression coefficients and alleviate the problems associated with separation.

**Conclusions:**

The current evidence supporting EPV rules for binary logistic regression is weak. Given our findings, there is an urgent need for new research to provide guidance for supporting sample size considerations for binary logistic regression analysis.

## Background

The number of subjects in the smaller of two outcome groups (‘number of events’) relative to the number of regression coefficients estimated (excluding intercept) has been identified as a key factor in the performance of binary logistic regression models [1–3]. This ratio is known as Events Per Variable (EPV). Earlier studies have demonstrated that the associations between covariates and the outcome estimated by logistic regression are often imprecise and biased in the direction of more extreme values when EPV is low [4–6]. Similarly, prediction models built using logistic regression in small data sets lead to poor predictions that are too extreme and uncertain [1, 3, 7, 8]. Ten EPV is a widely adopted minimal guideline criterion for performing binary logistic regression analysis [9–11].

Despite the wide acceptance of the minimal 10 EPV rule in medical literature, the results of three well-known simulation studies examining the minimal EPV criterion for binary logistic regression models are highly discordant [12–14]. These large differences in simulation results have in turn led to conflicting minimal EPV recommendations in these papers. Of these three studies, only Peduzzi et al. [12] supports the 10 EPV rule, after concluding that ‘no major problem occurred’ if EPV exceeds 10. In contrast, Vittinghoff and McCulloch [13] have argued that an EPV of 10 as a minimal guideline criterion is too conservative, showing that severe problems mainly occur in the EPV = 2 to EPV = 4 range. Conversely, Courvoisier et al. [14] showed that substantial problems may still occur ‘even if the number of EPV exceeds 10’. They showed that the performance of the logistic model may depend on various factors other than EPV, including the strength of associations between covariates and outcome and the correlation between covariates.

In this paper we offer explanations for the large differences between minimal EPV recommendations from previous simulation studies [12–14]. We focus on the accuracy of logistic regression coefficients (i.e., logit coefficients) in low EPV settings. Two issues are known to complicate the interpretation of logit coefficients in this setting. First, the estimation of logit coefficients by maximum likelihood is sometimes inaccurate when EPV is low. Second, ‘separation’ is likely to occur in low EPV settings. When separation occurs, the maximum likelihood estimation fails. We first briefly discuss each of these two issues.

### Accuracy of logit coefficients in small samples

In a typical binary logistic regression analysis, the strength of associations between covariates and outcome are quantified by the logit coefficients, which are estimated by maximum likelihood. While these estimators of the (adjusted) log-odds ratio have attractive asymptotic properties (e.g., unbiasedness and normality), these properties do not to apply in small samples. For example, the logit coefficients suffer from small sample bias [4, 5], leading to systematically overestimated associations. Also, asymptotic confidence intervals often do not have nominal coverage rates in studies with small data sets [12, 15]. Both problems are expected to become less likely with increasing sample size and increasing EPV.

The inaccuracies in the coefficients and corresponding confidence intervals lead to inaccurate inferences about the true covariate-outcome associations. Hereafter we refer to these problems as ‘inaccuracy in logit coefficients’.

### Separation

**Fig. 1:**
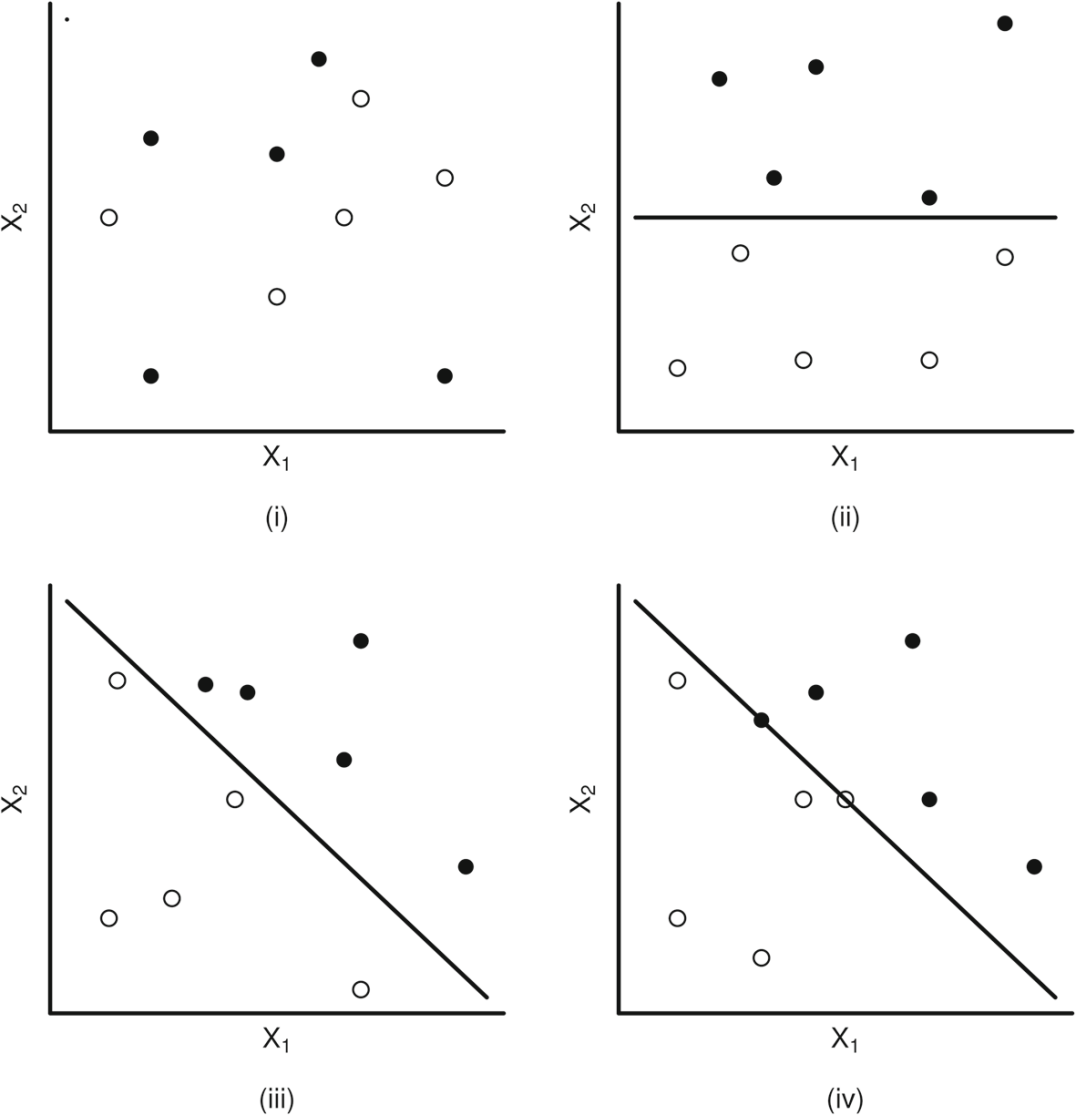
Graphical representation of separation (complete and quasi-complete) adapted from Albert and Anderson [16]. Sample points for two variables X_1_ and X_2_ by outcome (*Y*): open and filled *circles* represent different levels of the outcome (*Y*=0 or 1). (**i**) No separation; (**ii**) complete separation by variable X_2_; (**iii**) complete separation by variables X_1_ and X_2_; (**iv**) quasi-complete separation by variable X_1_ and X_2_

Another source of difficulty occurs when a single covariate or a linear combination of multiple covariates perfectly separates all events from all non-events [16, 17]. This phenomenon is referred to as ‘separation’ or ‘monotone likelihood’ (illustrated in Fig. 1). Estimating a logistic regression model by maximum likelihood on a ‘separated data set’ leads to non-unique point estimates and standard errors of coefficients near the extremes of parameter space [18]. On separated data, convergence of the iterative maximum likelihood estimation procedure may sometimes not be achieved as the upper bound on the number of iterations is reached (‘non-convergence’). Or, the solution may converge to a point that is not the maximum likelihood [16]. Because convergence criteria will often differ between software programs, estimates can vary considerably between software programs when fitting a logistic model on separated data.

The probability of separation occurring increases with decreasing sample size and increasing number of covariates. Hence, separation is likely to occur in low EPV data sets. In simulation studies, including those that examined the minimal EPV criterion for binary logistic regression, the occurrence of separated data sets has typically been treated as a nuisance. Researchers remove the simulation data set when separation is detected. Doing so, however, a non-random subset of simulated data sets is missing when analyzing the simulation results: particularly those data sets with strong associations between the covariates and the outcome [19]. The approaches to identify and handle separated data may therefore strongly affect the results and inferences of simulation studies.

### Outline of the paper

In simulation studies involving small samples and low EPV, some degree of inaccuracy in logit coefficients and separation is likely to coexist. Simulation results will therefore reflect the net effect of inaccurate estimation and handling of separated data sets. To gain insight into both problems separately, we will first investigate the factors driving the accuracy of logit coefficients by examining scenarios in which drawing separated data sets is highly unlikely (part I). Next, we examine a range of scenarios in which the probability of drawing a separated data set is substantially larger than zero (part II). In part II, we monitor the variations in simulation results due to different approaches of detecting and handling separated data sets. In both parts we will expore whether a simple-to-apply penalized estimation procedure suggested by Firth [17, 20] in combination with profile likelihood based confidence intervals can effectively improve the accuracy of logit coefficients in small samples. In the discussion, we will return to the differences in results of the previous minimal EPV simulation studies [12–14] using the findings from our simulations.

## Methods

### General

For each simulated data set, *N* covariate vectors *X*_1_,…,*X*_*P*_ were drawn from either an independent multivariate normal distribution (in part I and part II) or an independent Bernoulli distribution (in part II). The outcome variable (*Y*) for each covariate vector was generated from a Bernoulli distribution with a covariate vector specific probability derived by applying the logistic function using the true values of the data generating model on the simulated covariate data. The data generating models only included first order covariate (main) effects, thus were of the form: logit(*Y*)=*β*_0_+*β*_1_ *X*_1_+…+*β*_*P*_ *X*_*P*_.

On each generated data set we fitted the logistic regression model by maximum likelihood that had the same form as the data generating model (i.e., fitting the correctly specified logistic regression model). We also applied the modified score equations procedure suggested by Firth [20] that removes a portion of the small sample bias that can be anticipated in the maximum likelihood estimates, by introducing a penalty on the likelihood. The penalty function is a Jeffries invariant prior [20]. Another advantage of Firth’s correction is that its coefficients, $\hat {\beta }_{1}^{F},\ldots,\hat {\beta }_{P}^{F}$, are finite even when estimated on a data set that is separated.

We examined the empirical distribution of the estimator of one of the regression coefficients, arbitrarily taking the coefficient for the first covariate (hereafter referred to as the primary coefficient), $\hat {\beta }_{1}$. Based on guidance by Burton et al. [21], we calculated the following quantities: i) bias in the primary coefficient, defined by: $\bar {\hat {\beta }}_{1}- \beta _{1}$, where $\bar {\hat {\beta }}$ is the arithmetic mean of $\hat {\beta }_{1}^{ML}$ or $\hat {\beta }_{1}^{F}$ over all simulated data sets; ii) relative bias in the primary coefficient, defined by $(\bar {\hat {\beta }}_{1}- \beta _{1})/\beta _{1}$, iii) coverage of the 90% confidence interval by calculating for each data set the Wald confidence interval by $\hat {\beta }_{1}^{ML} \pm 1.645 \times \text {SE}(\hat {\beta }_{1}^{ML})$, where SE$(\hat {\beta }_{1}^{ML})$ is the estimated (ML) standard error for $\hat {\beta }_{1}^{ML}$. For $\hat {\beta }_{1}^{F}$ we estimated the profile likelihood 90% confidence interval [18]; iv) average 90% confidence interval width, defined by average of the difference between the upper and lower bounds of the 90% confidence intervals; v) mean square error (MSE): $(\bar {\hat {\beta }}_{1}- \beta _{1})^{2}+(\text {SD}(\hat {\beta }_{1}))^{2}$, where SD$(\hat {\beta }_{1})$ is the standard deviation of $\hat {\beta }_{1}^{ML}$ or $\hat {\beta }_{1}^{F}$ over the simulation data sets.

### Simulation procedures

In total, 465 different simulation scenarios were examined. For each of these scenarios, 10,000 data sets were generated using R software version 3.1.1 [22]. For each data set, sampling was continued until the prespecified criteria for sample size and the number of events were met, keeping the first events and non-events generated up to the required number of each. This procedure ensured a fixed sample size (*N*) and number of events (EPV) in each data set. This approach, which is equivalent to the approach used by Vittinghoff and McCulloch [13], takes advantage of the properties of the logistic model where only the intercept is affected by this sampling procedure.

The logistic regression models fitted by maximum likelihood and Firth’s correction were implemented using the glm function in the stats library (version: 3.1.1) and the logistf function in the logistf library (version: 1.21), respectively. To identify separation of simulation data sets the maximum likelihood standard errors of parameters were monitored through a re-estimation process [23]. This procedure is explained in detail in the Appendix. Unless otherwise specified: the default software criteria for convergence were used, calculation of the regression coefficient accuracy measures were based only on converged simulation results and maximum likelihood estimates for data sets that exhibited separation were excluded from the calculation of simulation results.

### Part I: Accuracy of logit coefficients

A series of scenarios were set-up to identify the factors that are driving the accuracy of the logit coefficient. In this first part we limited ourselves to scenarios in which the probability of drawing a separated data set was close to zero (maximum percentage separated data sets in a single simulation scenario of 0.3%; zero separated data sets in 98% of scenarios). To keep the probability of drawing a separated data set low, covariate data were sampled only from continuous (multivariate normal) distributions. Part I was further subdivided into four small-scale factorial simulation studies (Ia to Id). In study Ia, the role of EPV and the true value of *β*_1_ on accuracy of logit coefficients was studied for the case of a single continuous covariate. The role of the number of covariates (*P*) was evaluated in study Ib. In study Ic, the role of the sample size was examined, reflecting the effect of increasing the number in the largest group (non-events). The role of covariate correlations was studied in study Id. Details of these four studies are summarized in Table 1.

**Table 1:** Design factorial simulation studies Ia to Id

	Study			
Factors	Ia	Ib	Ic	Id
Sample size				
EPV (with steps of)	15 to 150 (5)	15 to 150 (5)	6 to 30 (2)	6 to 30 (2)
Outcome prevalence	1/2	1/2	1/2,1/3,1/4,1/5,1/10	1/4
Range sample size	30 to 300	60 to 1200	24 to 600	60 to 300
Effect size				
Value of $e^{\beta _{1}}\phantom {\dot {i}\!}$	1/4, 1/2, 1, 2, 4	2, 4	2	2
Value of $e^{\beta _{j}}, j > 1\phantom {\dot {i}\!}$	Not applicable	*β*_1_=…=*β*_*P*_	2	2
Covariates				
Number (*P*)	1	2, 3, 4	2	2
Distribution	(Multivariate) standard normal			
Correlation	Not applicable	0	0	.1,.15,.2,.25

### Part II: Detection and handling of separated data sets

In part II we evaluated the impact of different approaches for the detection and handling of separated data sets on simulation results and inferences. Two different simulation studies were conducted, which are explained below.

#### IIa. Binary single covariate

In study IIa, we investigated the extent to which simulation results differ between using all simulated data sets (a naive approach, using the software output regardless of convergence status) versus removing all separated data sets for quantifying the accuracy of logit coefficients. We also explored how the simulation results in terms of bias are affected by replacing the results of separated data sets by the highest estimated coefficient on non-separated data (an ad-hoc approach). Data were sampled for a single binary covariate with probability of sampling either observation of.5. The manipulated factors were: EPV and the true value of *β*_1_. We considered EPV values between 6 and 30, at incremental steps of size 2 and the values of the primary coefficient (*β*_1_) were chosen as log(1), log(2) and log(4).

#### IIb. Single simulation scenario, continuous covariate

In study IIb, we evaluated the impact of using different methods to detect the presence of separated data sets. In the first approach we used likelihood non-convergence as a criterion for removing simulation data sets, as was done in previous studies [12, 13]. This type of non-convergence occurs when the tolerance convergence criterion is not met while the upper bound on the number of iterations is reached. We compare this convergence criterion to our (computationally intensive) method of separation detection (see Appendix), and to the method used by Courvoisier [14]: a simulation data set is removed if for any parameter $j \neq 0, |\hat {\beta }_{j}|$ >log(50). To evaluate the effect of changing the likelihood criterion, four additional criteria for convergence tolerance (tol) and maximum number of Fisher scoring iterations (max-iter) are used: tol: 1e-8, max-iter: 25 (glm function default), tol: 1e-6, max-iter: 25 (Type I), tol: 1e-10, max-iter: 25 (Type II), tol: 1e-10, max-iter: 50 (Type III). Univariate covariate data were generated from standard normal distribution, the ratio of events to non-events was kept constant at 1:1. EPV was fixed at 4 and *β*_1_= log(4).

## Results

### Part I: Accuracy of logit coefficients

**Fig. 2:**
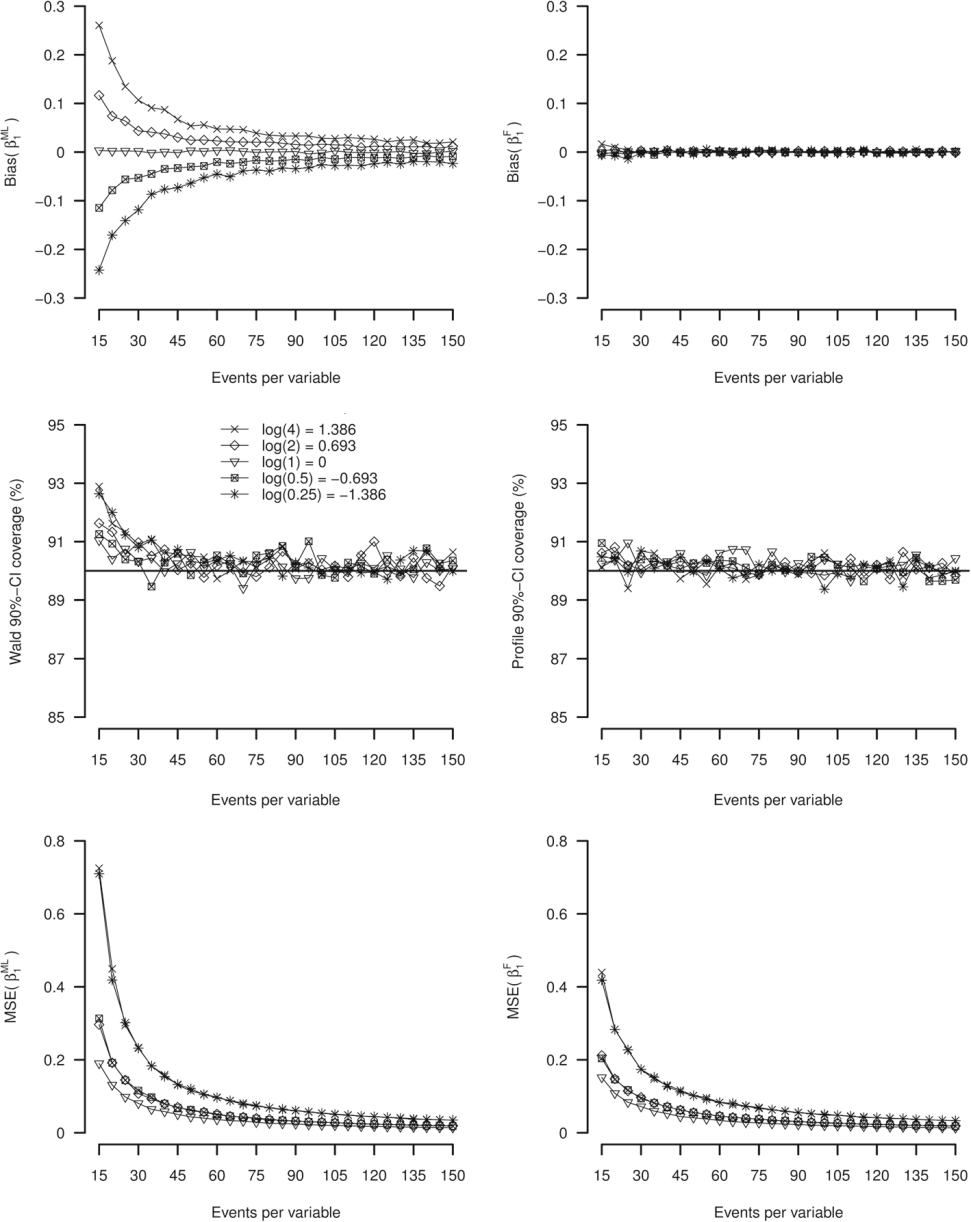
Results of simulation study Ia. Accuracy as a function of EPV and true value of the log-odds ratio (*β*_1_). *Left panel*: maximum likelihood logistic regression, *right panel*: Firth’s correction

Figure 2 shows the simulation results for study Ia. With traditional logistic regression (upper left panel), for true non-zero covariate-outcome associations the primary logit coefficient ($\beta _{1}^{ML}$) was biased towards more extreme values (away from zero). Bias decreased with increasing EPV through a non-linear function (that can be approximated by: $\text {log}(|\text {bias}(\beta _{1}^{ML})|) = \lambda _{0} - \lambda _{1} \text {log(EPV)}$, where *λ*_0_>0 and *λ*_1_>0, for which the values depend on the simulation setting). Bias in the logit coefficient did not reduce strictly to zero even for EPV as large as 150. Bias depended on the true effect size of the coefficient with bias increasing in case of stronger associations. The figure further illustrates that bias is symmetric but in opposite directions for the conditions with the same true effect size (the effect of recoding the outcome variable: such that *β*= log(2) becomes *β*=log(1/2) and *β* = log(4) becomes *β*=log(1/4), or vice versa). Bias in Firth’s estimator (${\beta _{1}^{F}}$, upper right panel) was close to zero for all studied EPV values and across all true effect sizes.

The middle left panel in Fig. 2 shows slight over-coverage of the 90% Wald-confidence interval for EPV <30. The profile likelihood confidence interval for Firth’s estimator, however, was close to the nominal level for all studied conditions. The mean square error of $\beta _{1}^{ML}$ and ${\beta _{1}^{F}}$ decreased with true effect size and EPV. The mean square error for ${\beta _{1}^{F}}$ was systematically lower than for $\beta _{1}^{ML}$.

**Fig. 3:**
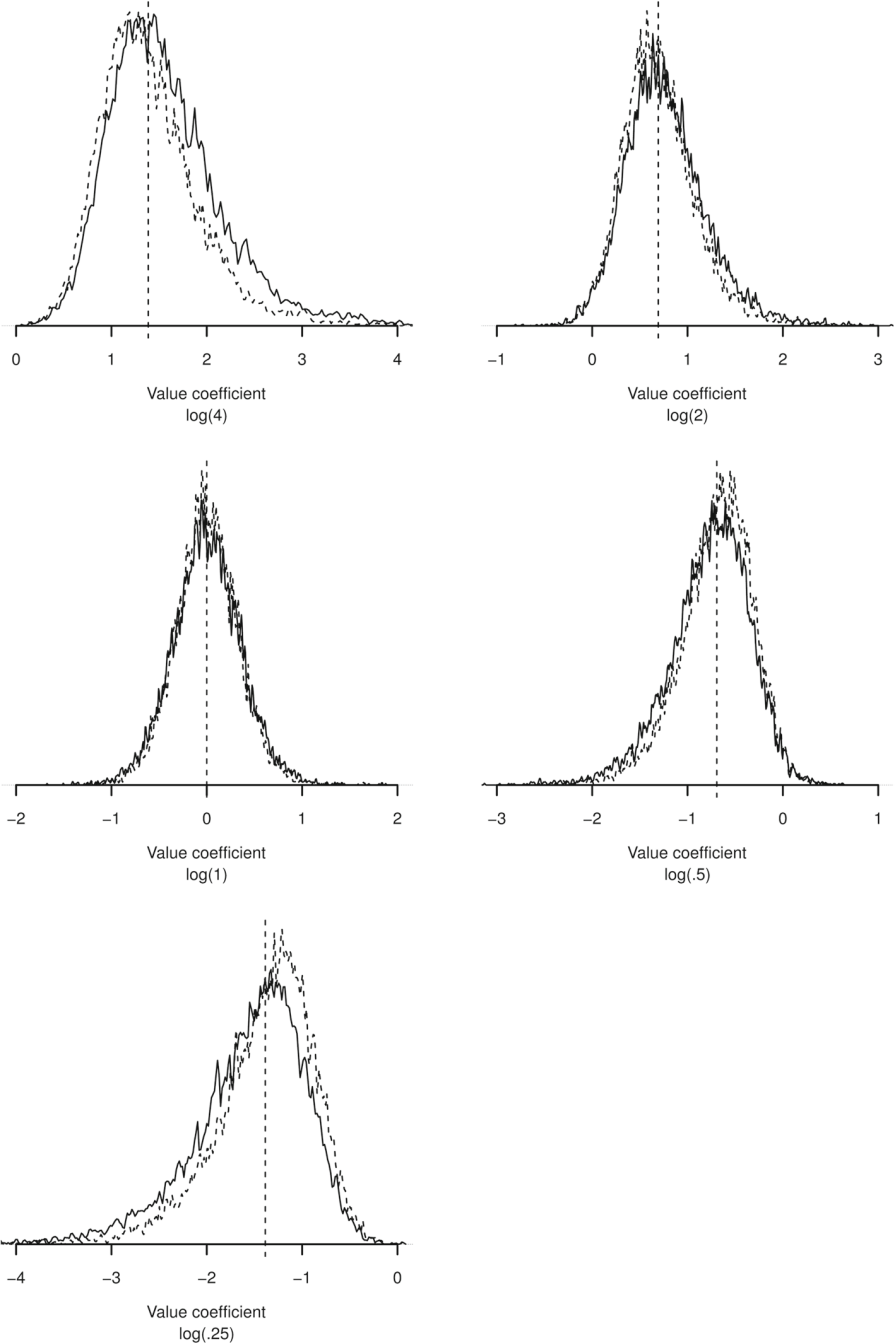
Density of estimated coefficients in simulation at EPV = 20 (study Ia) for different true values of the log-odds ratio. *Vertical dashed line* is true value of the regression coefficient. *Solid line*: maximum likelihood logistic regression; *dashed line*: Firth’s correction

The empirical sampling distributions of $\hat {\beta }_{1}^{ML}$ and $\hat {\beta }_{1}^{F}$ at EPV = 20 (study Ia) are presented in Fig. 3. The sampling distributions show severe non-normality when the covariate-outcome associations are non-zero. The degree of non-normality increased with true effect size. The effect of Firth’s correction is illustrated by comparing the distribution of $\hat {\beta }_{1}^{F}$ estimates to the $\hat {\beta }_{1}^{ML}$ distribution: the $\hat {\beta }_{1}^{F}$ estimates were shrunken towards zero; the magnitude of shrinkage was proportional to the estimated effect size. The arithmetic mean of the $\hat {\beta }_{1}^{F}$ distribution for a non-zero true association was closer to zero and the long tail (tail in the direction of stronger effect size) was smaller.

**Fig. 4:**
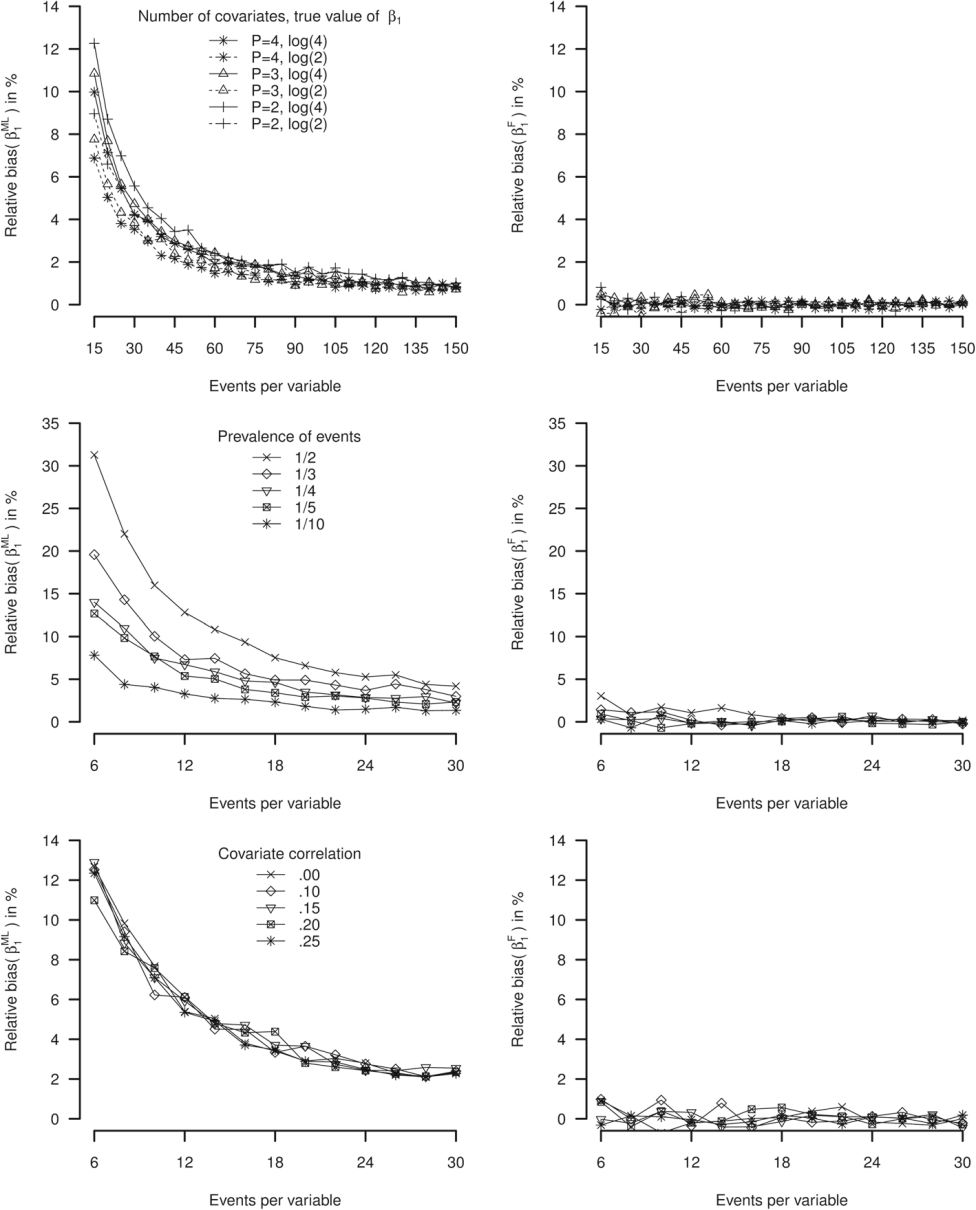
Relative bias simulation studies Ib, Ic, and Id. *Left panel*: maximum likelihood logistic regression, *right panel*: Firth’s correction

Figure 4 shows the relative bias under varying number of covariates (study Ib), sample size (study Ic) and covariate correlation settings (study Id). The maximum likelihood estimates were always biased away from zero. Bias decreased with the addition of more covariates and was affected by the size of the true effect (Fig. 4, upper panel) and the total sample size (Fig. 4, middle panel). There was no apparent effect on bias by varying the correlation between covariates in the model (Fig. 4, lower panel). In each study and each simulation condition, ${\beta _{1}^{F}}$ was a close to unbiased estimator.

Table 2 summarizes the results for the four factorial simulation studies. Average bias and average mean square error decreased with increasing EPV in case of maximum likelihood estimates. Average coverage for the maximum likelihood Wald confidence interval based and Firth’s correction profile likelihood confidence intervals were close to nominal (90%) in most situations, with a small over-coverage in lower EPV settings (though not exceeding 93%). The average width of the confidence intervals and mean squared error were systematically smaller after applying Firth’s correction.

**Table 2:** Results simulation studies Ia to Id

Study	Study Ia^*^ and Ib						Study Ic and Id					
EPV	15 to 30		35 to 50		55 to 150		6 to10		12 to 18		20 to 30	
Estimator	$\beta _{1}^{ML}$	${\beta _{1}^{F}}$	$\beta _{1}^{ML}$	${\beta _{1}^{F}}$	$\beta _{1}^{ML}$	${\beta _{1}^{F}}$	$\beta _{1}^{ML}$	${\beta _{1}^{F}}$	$\beta _{1}^{ML}$	${\beta _{1}^{F}}$	$\beta _{1}^{ML}$	${\beta _{1}^{F}}$
Bias												
Average bias	0.084	0.002	0.038	0.001	0.016	0.000	0.069	0.002	0.033	0.000	0.020	0.000
max	0.261	0.016	0.091	0.005	0.056	0.006	0.217	0.021	0.075	0.011	0.046	0.005
min	0.025	-0.004	0.013	-0.002	0.004	-0.005	0.023	-0.005	0.016	-0.003	0.009	-0.003
Average relative bias (%)	7.8	0.1	3.6	0.1	1.5	0.0	8.4	0.4	4.8	0	2.9	0
max	18.8	1.2	6.6	0.5	4.0	0.5	31.2	3.0	10.8	1.6	6.5	0.7
min	3.5	-0.5	1.9	-0.3	0.5	-0.7	3.3	-0.7	2.3	-0.5	1.3	-0.0
>+10% relative bias (%)	18.8	0	0	0	0	0	37.5	0	3	0	0	0
Coverage 90% CI												
Average coverage (%)	90.4	90.1	90.2	90.2	90.1	90.0	90.4	90.3	90.2	90.2	90.1	90.2
max	92.9	90.8	91.1	90.7	91.0	90.7	92.1	91.2	90.8	90.6	90.9	90.8
min	89.1	89.4	89.3	89.6	89.4	89.2	89.6	89.6	89.7	89.6	89.3	89.6
> ± 1% nominal (%)	15.6	0	3.1	0	0.6	0	10	2.5	0	0	0	0
Average width	1.102	1.059	0.752	0.738	0.487	0.483	1.183	1.133	0.828	0.811	0.653	0.646
Mean Square Error												
Average MSE	0.160	0.118	0.063	0.055	0.025	0.024	0.169	0.125	0.070	0.062	0.042	0.039
Separated data sets												
Total (%)	0.006		0		0		0.001		0		0	

^*^only for *β*_1_≥*l* *o* *g*(1)

### Part II: Detection and handling of separated data sets

**Fig. 5:**
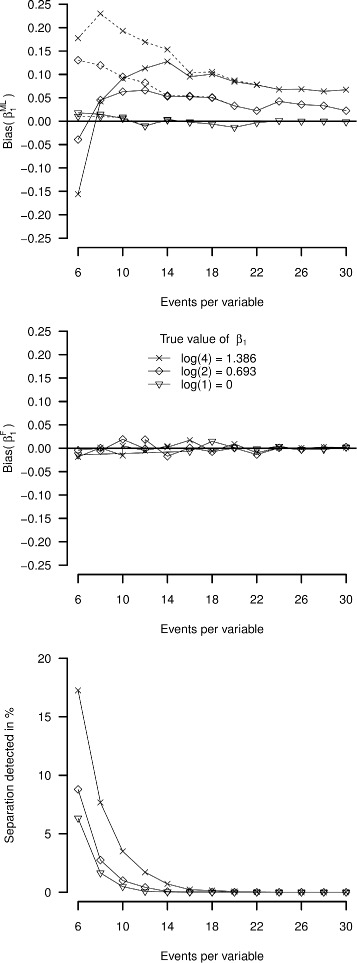
Simulation study IIb results. *Upper panel, solid line*: data sets removed from analysis; *Upper panel, dashed line*: data sets replaced by maximum non-separated effect size. *Middle panel*: Firth correction. *Lower panel*: percentage of separated data sets by true effect size

The results for study IIa are given in Table 3 and Fig. 5. In Table 3 the simulation results were calculated twice, once by removing the separated data sets from analysis and once by leaving the separated data sets in, using the estimates at the point at which the model had converged (in case of covergence) or the estimate at the point that is the maximum number of iterations (in case of non-convergence). Between these approaches the calculated bias and MSE for EPV values between 4 and 18 were noticeably different. Average coverage in those EPV ranges was not markedly different, while average width of the confidence interval differed strongly depending on the handling of separated data sets. For EPV values between 55 and 150, separation was detected just eight times. In these simulations, only the calculated average width of the confidence interval and, to a lesser extent, mean square error were different between the two approaches of handling the separated data sets.

**Table 3:** Results simulation study IIa, maximum likelihood logistic regression only

EPV	15 to 30		35 to 50		55 to 150	
Separated data removed	Yes	No	Yes	No	Yes	No
Bias						
Average bias	-0.097	2.255	0.083	0.161	0.051	0.053
max	0.091	7.074	0.127	0.439	0.084	0.096
min	-0.556	0.234	0.050	0.056	0.048	0.022
Average relative bias (%)	-0.087	2.110	0.079	0.145	0.048	0.049
max	0.091	5.103	0.095	0.317	0.061	0.069
min	-0.401	0.338	0.069	0.081	0.032	0.032
Coverage 90% CI						
Average coverage (%)	92.7	93.4	89.1	89.1	90.4	90.4
max	98.3	98.8	90.6	90.6	91.8	91.8
min	89.7	89.8	87.9	87.9	89.2	89.2
> ± 1% nominal (%)	75	75	50	37.5	25	25
Average width	4.087	4437.2	2.656	49.2	2.005	2.645
Mean Square Error						
Average MSE	1.251	64.571	0.709	2.243	0.397	0.422
Separated data sets						
Total (%)	13.2		4.2		0.006	

In the lower panel of Fig. 5 it can clearly be seen that separation of the simulation data sets was rare for EPV values of 18 or higher. For these scenarios, bias in the maximum likelihood estimates (upper panel) for the non-zero true associations decreased with increasing EPV. For an EPV values of 16 and lower, separation occurred more frequently. The likelihood of drawing separated data sets also increases with true effect size of the coefficient. When removing those data sets from the analysis (upper panel, solid line), for the non-null associations the bias is underestimated, and even becomes negative at EPV values of 6 and 8. When replacing the results for the separated data sets by the highest estimated effect sizes (dashed lines, upper panel), the simulation outcomes are more in line with the patterns we observed in Part I. Finally, using Firth’s correction (Fig. 5, middle panel) all data sets were analyzed and the relative bias was near zero across the whole range of EPV.

The results for study IIb are shown in Table 4. In this single scenario study, the prevalence of separated data sets was 5.8% (as detected through the preferred re-estimation process, see Appendix). The differences in the calculated simulation results between the six methods of separation detection and estimation were large. Differences were noticeable especially in the calculated (relative) bias, mean square error and width of confidence intervals. Coverage was not significantly affected across the 6 approaches to detect separation. The success rate of using convergence as a criterion to detect separation depended on the convergence criteria. Relying on the Type III convergence criterion (only.09% non-convergence) makes the simulation results non-interpretable. The use of $|\hat {\beta }_{j}^{ML}|$ >log(50) as a separation criterion in this scenario shows very different results compared to our preferred re-estimation method to detect separation.

**Table 4:** Results simulation study IIb

Estimator	${\beta _{1}^{F}}$	$\beta _{1}^{ML} $	$\beta _{1}^{ML} $	$\beta _{1}^{ML} $	$\beta _{1}^{ML} $	$\beta _{1}^{ML} $	$\beta _{1}^{ML} $
Separation detection	NA	Tracing^b^	Estimate^c^	None	None	None	None
Convergence criterion^a^	Default	Default	Default	Default	Type I	Type II	Type III
Data sets removed (%)	0	8.06	16.64	5.12	0.34	6.29	0.09
Bias	0.012	0.569	0.186	1.672	17.5	0.856	41.3
Coverage 90% CI	0.919	0.949	0.937	0.944	0.947	0.944	0.947
Mean width 90% CI	4.32	4.50	3.64	5018	13620	6.03	1135784
MSE	1.080	2.681	0.904	71.563	11532	319	173726

^a^default: tol: 1e-8, max-iter: 25, Type I: tol: 1e-6, max-iter: 25, Type II: tol: 1e-10, max-iter:25, Type III: tol: 1e-10, max-iter:50

^b^criterion: re-estimation process, variance of scaled standard errors >20 (see Appendix)

^a^criterion: if for any parameter $j \neq 0, |\hat {\beta }_{j}|$ >log(50)

## Discussion

This paper offers explanations for the large differences between minimal EPV recommendations from previous simulation studies [12–14]. EPV, which is thought to be a key determinant of the performance of logistic regression models, is frequently used in sample size considerations and as a methodological quality item for critically appraising published studies [9–11]. To explain the differences in minimal EPV recommendations we distinguished between two small sample issues that coexist in the earlier studies, namely: biased estimation of logit coefficients and the problem of separation. While biased estimation of coefficients is often of primary interest, separated data sets are an important nuisance. The approach to detect and handle separation has a strong impact on the results. We now discuss separately: i) the drivers of the accuracy of logit coefficients; ii) the influence of separated data sets on simulation results; iii) reasons for large differences between the earlier minimal EPV simulation studies.

### Drivers of the accuracy of logit coefficients

Our results show that logit coefficients are typically overoptimistic estimates of the true associations when estimated by maximum likelihood in small to moderated-sized data sets. This over-optimism is commonly referred to as finite sample bias [24], and is well described in statistics literature [3, 7]. The bias can to a large extent be attributed to skewed sampling distributions of the estimator in small data. Our simulations show that the finite sample bias is larger for data sets with small EPV, and may not reduce strictly to zero even for an EPV of 150. In simulations where by design separation of data sets occurred only rarely, we found that bias depends on various factors besides EPV, notably, the true (multivariable) effect size of the regression coefficient. This latter finding is to be expected, based on the analytical work of Cordeiro and McCullagh [25]. Further, we showed that bias can be reduced by increasing the total sample size while keeping EPV constant (i.e., increasing the number of non-events). Bias at a fixed value of EPV also decreases with the number of covariates included. For a few conditions, we found that the Wald confidence interval showed slight over-coverage at smaller values of the EPV, i.e., for EPV <30 in the case of a single covariate. We could find no evidence to support that the correlation between covariates in the model affected the accuracy of the coefficients as previously suggested [14].

Our study further suggests that Firth’s correction [20] can reduce finite sample bias close to zero and reduce mean square error. Profile likelihood confidence intervals for the Firth’s corrected estimates showed close to nominal behavior, and on average have smaller width than the traditional Wald confidence interval for the maximum likelihood estimates. Firth’s correction is one of several methods for increasing the efficiency of the estimators in logistic regression with small samples [11, 15]. In particular, these alternatives seem beneficial for analyzing data sets with sample sizes in the order of a few hundreds. Procedures implementing Firth’s correction for logistic regression (and Cox regression) are available in many statistical software packages (such as SAS, Stata and R).

### The impact of separated data sets on simulation results

The traditional (maximum likelihood) logistic regression analysis of a dataset in which the included covariates perfectly separate the binary outcome variable cannot be trusted. In such cases, typically, very low or very high parameter estimates with large maximum likelihood standard errors are returned by the statistical software program. The estimated values, however, are rather arbitrary and depending on software settings such as likelihood convergence criteria. In the context of simulation studies these ‘extreme’ values can have a large influence.

Methods to detect separation in simulation studies can be computationally intensive [23, 26] and likely therefore not routinely applied in most simulation studies. We also showed that convergence as a criterion for separation detection often fails. Separated data sets may therefore often remain undetected.

If separation is detected, the common approach is to remove the results based on separated data sets from the analysis. Steyerberg et al. [19] recognized that this causes informative missingness of simulation results. Our simulations confirm that even when the proportion of separated data sets is relatively small (∼5%), removing separated data sets from analysis has a large impact on (apparent) bias, mean square error and width of the confidence intervals. Alternatively, replacing these results, for example by the ‘largest’ non-separated simulated effects, may be a more realistic approach. It must be recognized that the choice of the replacing value (or mechanism) is again rather arbitrary and may heavily influence the simulation results.

Separation of the outcome by covariates not only occurs in the setting of the binary logistic model. For example, separation can also occur with logistic regression for more than two outcomes and Cox’s proportional hazards regression [27, 28]. Reporting on the proportion of separated simulation data sets is, however, highly uncommon in simulation studies.

By applying Firth’s correction, the problems associated with separation can be avoided.

### Reasons for differences between EPV simulation studies

We identified two major reasons for the differences in results and recommendations between the preceding simulation studies [12–14]. First, differences in the design of the simulation studies may have contributed to variations in simulation outcomes at the same level of EPV. The preceding studies [12–14] differ, for example, in their range of simulated true effect sizes of the regression coefficient, total sample size and the number of included covariates. Second, none of these studies have sufficiently addressed the issue of separated simulation data sets. We illustrated that separated data sets can lead to misleading simulation outcomes. As separated data sets occur most frequently in low EPV settings, these settings are likely most affected.

The probability of drawing separated data in simulations depends on a multitude of factors, including the total sample size, the true effect sizes of the coefficients and the correlation between the covariates [17]. Developing simulation scenarios in realistic contexts where this probability is close to zero is difficult. For example, it was difficult to design small sample simulation settings with binary predictor variables while avoiding separation. Hence, in the setting of small EPV simulation studies, developing realistic full factorial simulation designs (i.e., a simulation design where all possible combinations of simulation factors are evaluated) in which the probability of drawing separated data sets in each condition is close to zero does not appear to be possible.

Steyerberg et al. [19] suggested the use of Firth’s correction as a method to perform minimal EPV simulation studies and we have shown that this solves the problem of separated data sets. However, due to the impact of Firth’s correction on the estimated coefficients even in the absence of separation, only little is learned about the behavior of traditional logistic regression analysis that is commonly used and is based on the generally well-trusted principles of maximum likelihood.

## Conclusion

We conclude that the evidence underlying the EPV = 10 rule as a minimal sample size criterion for binary logistic regression analysis is weak. So far, much of this evidence comes from minimal EPV simulation studies that studied the performance of estimating the relations between covariates and outcome. Our simulation study shows that this performance at low values of EPV can be significantly improved using Firth’s correction. In this paper we have not studied the impact of small samples in relation to number of covariates with respect to the model’s predictive accuracy (e.g. model calibration and discrimination). The studies by Steyerberg et al. [29] and Ambler et al. [30] give some insight and guidance. However, we believe that also in this area larger scale simulation studies are urgently needed to provide guidance for supporting sample size considerations for binary logistic regression analysis.

## Appendix

To detect separation in a data sets it is sufficient to monitor the maximum likelihood standard errors of parameters during the estimation process [23]. The logistic regression model is re-fitted on each simulation data set with 1,2,…,30 Fisher scoring iterations. The maximum likelihood standard errors for each of the 30 refits are collected. This approach to identification of separation is similar to the default method for separation detection in the brglm package (Version 0.5-9) for R by Ioannis Kosmidis. Separation for a parameter is said to occur if the variance of scaled standard errors (such that standard errors on first iteration equal 1) over refits was larger than 20. This cut-off value was chosen based on a small pilot study. Results not shown.

## Abbreviations

EPV: Events per variable
